# Solid-State rGO-PEDOT:PSS Transducing Material for Cost-Effective Enzymatic Sensing

**DOI:** 10.3390/bios9010036

**Published:** 2019-03-01

**Authors:** Firdaus Abd-Wahab, Habibah Farhana Abdul Guthoos, Wan Wardatul Amani Wan Salim

**Affiliations:** Department of Biotechnology Engineering, Faculty of Engineering, International Islamic University Malaysia, Gombak 50728, Kuala Lumpur, Malaysia; firdaus@iium.edu.my (F.A.-W.); habibahfarhana@gmail.com (H.F.A.G.)

**Keywords:** screen-printed carbon electrode, reduced graphene oxide, PEDOT:PSS, electrochemical reduction, glucose oxidase, cyclic voltammetry, surface characterization

## Abstract

Performance of a sensing device is dependent on its construction material, especially for components that are directly involved in transporting and translating signals across the device. Understanding the morphology and characteristics of the material components is therefore crucial in the development of any sensing device. This work examines the morphological and electrochemical characteristics of reduced graphene oxide interspersed with poly(3,4-ethylenedioxythiophene):poly(styrenesulfonate) (rGO-PEDOT:PSS) used as a transducer material deposited on a commercially available screen-printed carbon electrode (SPCE). Electron microscopy shows that PEDOT:PSS is interspersed between rGO layers. Raman and XRD analyses suggest that the graphene crystallinity in GO-PEDOT:PSS and rGO-PEDOT:PSS remains intact. Instead, PEDOT:PSS undergoes a change in structure to allow PEDOT to blend into the graphene structure and partake in the π-π interaction with the surface of the rGO layers. Incorporation of PEDOT:PSS also appears to improve the electrochemical behavior of the composite, leading to a higher peak current of 1.184 mA, as measured by cyclic voltammetry, compared to 0.522 mA when rGO is used alone. The rGO-PEDOT:PSS transducing material blended with glucose oxidase was tested for glucose detection. The sensitivity of glucose detection was shown to be 57.3 µA/(mM·cm^2^) with a detection limit of 86.8 µM.

## 1. Introduction

Screen-printed electrodes allow for fabrication of miniaturized and portable electrochemical devices. Among the different types of such electrodes, various portable devices have adopted the use of a screen-printed carbon-based electrode owing to its low production cost [1]. This permits the development of affordable disposable electrodes to be used in electrochemical sensors. 

However, affordability of the screen-printed carbon electrode (SPCE) comes with its own limitations. Direct use of the SPCE without any modifications restricts its sensitivity and selectivity towards the electron-transfer reaction occurring at the electrode-electrolyte interface [2]. The electrode surface therefore requires some modification to increase its electrochemical activity and sensitivity. Such modifications include improving signal transduction at the electrode-electrolyte interface, either by pre-treating the electrode surface [2,3] or by incorporating better conductive and electrocatalytic materials [4]. Selecting a conductive transducer layer requires an understanding of the mechanism driving the electronic or ionic transport across the material. The faster an electron is funneled through the material, the faster that electron is transferred at the electrode surface; the transfer rate is influenced by the electrochemical characteristics of the material.

Graphene, along with its derivatives, has been diversely used and manipulated for its surface properties. Inclusion of graphene has been shown to improve the electrochemical performance of electrodes [5,6]. Surface functionalization of the SPCE, either with graphene or graphene oxide, affects how charge is transferred and detected. However, functionalization of an SPCE with graphene has commonly been performed by using graphene oxide [7]. Graphene oxide is usually drop cast followed by a reduction process that turns the film into a reduced-graphene oxide structure almost like that of graphene. While chemical reduction is still used, electrochemical reduction of graphene oxide is much preferred for its rapidity and non-toxicity [8]. 

In developing enzymatic biosensors, construction of the transducer layer also needs to take into consideration the biorecognition element. Detection of biologics is highly dependent on the specificity and selectivity of the biorecognition element [9]. Recognition then sends an electrical or chemical signal that needs to be recognized and propagated at the transducer layer for the signal to be detected. The material used in the transducer layer should be able to accommodate and transduce the signal from the biorecognition element [10,11]. Measuring the signals could then be done by either impedimetric or potentiometric means; these methods of detection are commonly used in electrochemical sensor development.

Conducting polymers comprise a group of polymeric materials that, instead of being insulative, can accommodate the propagation of electrons and/or ions. Owing to this distinct characteristic, conducting polymers are sought after for use in electronic and solar applications [12]. Inclusion of conducting polymers has been shown to improve power-conversion efficiency of solar cells [13,14]. The use of conducting polymers as transducers has also been reported to improve the conductivity of electrodes. It has opened up the possibility of developing sensors based on solid-state electrodes [15]. One particular polymer of interest to us is a thiophene-based composite, poly(3,4-ethylenedioxythiophene):poly(styrenesulfonic acid), or PEDOT:PSS. This polymer has been reported to have high conductivity with low oxidation potential and low bandgap [16], which makes it an attractive choice in electrochemical sensors [17,18]. PEDOT:PSS is also regarded as an environmentally friendly and easily processable material [16,19], favorable factors for device development and mass production [17,20].

Incorporation of either conductive graphene or reduced graphene oxide into PEDOT:PSS has been described to help stabilize the transport layers of the polymer [13,14]. It is also reported to improve flexibility and capacitance of the composite when formed into free-standing films [21]. Yet, the contributing factor that we believe most motivates the use of reduced graphene oxide and PEDOT:PSS is the low cost of these materials for device development [14,22,23,24]. Most of the studies that use reduced graphene oxide with PEDOT:PSS, however, appear to reduce the graphene oxide either chemically or thermally. In this study, we reduced the graphene oxide electrochemically on the SPCE. The reduced-graphene oxide and PEDOT:PSS mixture could be directly deposited onto the surface of the transducer, which allowed for rapid prototyping and scaling-up without the need of a clean room. 

Many papers discuss the applications of the composite of graphene and PEDOT:PSS, but few efforts have been made to understand the composite structure, redox behavior, surface characteristics, and feasibility as enzymatic sensors. The catalytic activity of enzymes is affected by their covalent and non-covalent interaction with an electrode material. Immobilization of enzymes on electrodes also affects its structural conformation, which in turn changes the immobilized enzymes’ catalytic activity. The interaction between graphene and graphene oxide and biomolecules is reviewed elsewhere [25,26]. These reviews emphasize the complicated nature of the interaction between peptides and graphene or graphene oxide, and most studies rely on experimental methods to understand the interaction. One study used electrochemical polymerization of EDOT to encapsulate glucose oxidase into the PEDOT structure, thus preserving enzyme structure and catalytic capability [27]. However, the aforementioned study of encapsulating glucose oxidase with PEDOT:PSS was conducted using tubular electrodes, and sensitivity was improved with the addition of gold metal. Our fabrication process started with drop-casting a premixed solution consisting of glucose oxidase, PEDOT:PSS, and graphene oxide on SPCEs and subsequently reducing the drop-cast graphene electrochemically. This simple fabrication process was tested for glucose sensing to understand the limitations of the fabrication process. Studies to understand the interaction of glucose oxidase as the model enzyme with the composite are beyond the scope of this work. A simple fabrication process reduces cost and allows rapid scale-up, but the fabrication process could affect enzyme catalytic activity.

In this study, we focus on examining the characteristics of reduced graphene oxide (rGO) in a composite with PEDOT:PSS as the transducer material on SPCEs. While characterization of graphene-PEDOT:PSS has been reported previously [28], studies on the feasibility of the material to modify SPCEs for electrochemical sensing are still lacking. Reduced graphene oxide has always been likened to graphene, but with defects [29]. These defects could possibly influence the interaction between rGO and PEDOT:PSS, as well as the SPCE surface. As a transducer material, rGO-PEDOT:PSS has the potential to improve signal transduction at the electrode-electrolyte interface for better detection.

## 2. Materials and Methods

### 2.1. Equipment

A potentiostat (PocketSTAT from IVIUM Technologies, Eindhoven, the Netherlands) was used for all electrochemical measurements and reduction processes. SPCEs with active electrode diameter Ø = 2 mm were purchased from Pine Instrument Company, Grove City, PA, USA. For surface characterization of the transducer material, SEM (AURA 100 benchtop, Seron Technologies, Uiwang-si, Gyeonggi-do, Korea), and Raman spectroscopy (uRaman-M, Avantes, the Netherlands) were conducted at the Centre of Advanced Materials, University of Malaya, Selangor, Malaysia. TEM (Tecnai TF20 X-Twin FEI, Thermo Fisher Scientific, Waltham, MA, USA) was done at the Malaysian Institute of Microelectronics Systems (MIMOS), BERHAD Technology Park, Seri Kembangan, Malaysia, FTIR spectrometer (Nicolet™ iS50, Thermo Fisher Scientific) at The International Institute for Halal Research and Training (INHART), IIUM, Selangor, Malaysia, and XRD (Bruker D8 Advance, Bruker Corporation, Billerica, MA, USA) at Universiti Kebangsaan Malaysia Research Centre, Bangi, Selangor, Malaysia.

### 2.2. Material and Reagents

Ultra-highly concentrated single-layer graphene oxide (UHC GO, 6.2 mg/ml) was purchased from Graphene Supermarket (Richmond, NY, USA). Poly (3, 4-ethylenedioxythiophene): polystyrenesulfonic acid (PEDOT:PSS, 483095) was purchased from Sigma-Aldrich, St. Louis, MO, USA. Potassium dihydrogen phosphate (KH_2_PO_4_, 795488) and disodium hydrogen phosphate (Na_2_HPO_4_, 71640) to prepare 0.1 M phosphate buffered saline (PBS), pH 5, were also obtained from Sigma-Aldrich. Potassium ferricyanide (K_3_Fe(CN)_6_, 6969-00) was purchased from R&M Chemicals, Selangor, Malaysia. Deionized (DI) water was used whenever required throughout experiments.

### 2.3. Preparation of rGO and rGO-PEDOT:PSS Electrodes

To prepare an rGO/SPCE, 3 µL UHC GO was drop-cast onto the working electrode (WE) of the SPCE and left to dry in ambient conditions. To prepare an rGO-PEDOT:PSS/SPCE, GO and PEDOT:PSS at a ratio of 1:1 were mixed, sonicated at 30 °C for 10 min, drop-cast onto the WE of the SPCE, and dried in ambient conditions. All drop-cast electrodes were reduced via electrochemical reduction using repetitive cyclic voltammetry (CV) for 15 cycles at scanning potential of 0 V to −1.5 V and scan rate of 0.1 V/s in 0.05 M PBS, pH 5.0 (disodium hydrogen phosphate/potassium dihydrogen phosphate; NaHPO_4_/KH_2_PO_4_). The modified electrodes were rinsed with deionized water and dried in ambient conditions. 

### 2.4. Surface Characterization

The morphologies of graphene and graphene-based nanocomposites were observed by SEM (AURA 100 benchtop) with a power setting of 5.0 kV and magnification settings of 10X. The Tecnai TF20 X-Twin FEI operating at an accelerating voltage of 200 kV was used to obtain TEM images of the samples. For the infrared spectra of graphene and graphene-based nanocomposites, a FTIR spectrophotometer (Nicolet™ iS50) was used, and the spectra were recorded in the range between 600 and 4000 cm^−1^, while the Raman spectra of the samples were recorded between 400 and 3000 cm^−1^ with a uRaman-M microscope using a 532-nm excitation wavelength. A Bruker D8 Advance instrument using Cu Kα radiation under a voltage of 40 kV and a current of 40 mA was used to perform XRD analyses, and the refraction data of the thin-film samples were recorded for 2θ angles between 5 and 60 degrees.

### 2.5. Cyclic Voltammetry of rGO/SPCE and rGO-PEDOT:PSS/SPCE

Cyclic voltammetry is an electrochemical analysis conducted when a potential voltage is applied to an electrode setup consisting of working and reference electrodes; a current response is measured using a potentiostat against a reference electrode—typically an Ag/AgCl electrode. CV was performed on the unmodified SPCE, rGO/SPCE, and rGO-PEDOT:PSS/SPCE in a redox-active solution of 0.1 M potassium ferricyanide (K_3_Fe(CN)_6_). This method was used to understand the electrochemical redox behavior of the material deposited on the WE surface. The oxidation/reduction peak current (I_p a/c_) of the electrode was determined by the Randles–Sevcik equation:

I_p a/c_ = (2.69 × 10^5^) *n*^3/2^D^1/2^ C A*v*^1/2^, (1)

where *n* is the number of transferred electrons for the redox reaction, D is the diffusion coefficient (6.70 × 10^−6^ cm^2^ s^−2^), C is the molar concentration of ferricyanide (0.1 M), A is the effective surface area (cm^2^), and *v* is the scan rate (mV s^−1^).

For the effective surface area of both modified and unmodified SPCEs, CV was performed at scan rates of 25, 50, 100, and 150 mV/s; from equation (1), a well-established linear relationship exists between I_p_ and *v*^1/2^. By performing linear regression for I_p_ versus *v*^1/2^, the slope *k* can be obtained, and one may express A as:

A = *k*/((2.69 × 10^5^) n^3/2^D^1/2^ C) (2)

## 3. Results and Discussion

### 3.1. Surface Morphologies and Characterization

**Figure 1:**
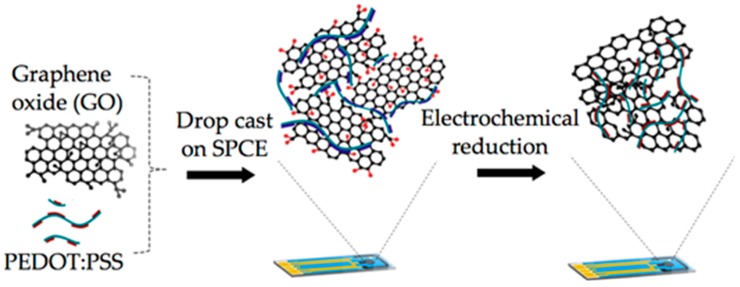
Schematic illustration of the modification done on the screen-printed carbon electrodes (SPCEs). Commercially available ultra-highly concentrated graphene oxide was initially mixed at a ratio of 1:1 with PEDOT:PSS before being drop-cast onto the working electrode of the SPCE and left to dry at room temperature, resulting in the formation of a GO-PEDOT:PSS/SPCE electrode. Subsequent electrochemical reduction would then form the rGO-PEDOT:PSS/SPCE electrode.

The fabrication process of the modified SPCE electrode is shown in Figure 1. The graphene oxide that was initially mixed with PEDOT:PSS was electrochemically reduced to convert the composite into rGO-PEDOT:PSS. The reduction process removed the oxygenated functional groups at the edges of the graphene oxide, thereby eliminating some of its insulative properties [29]. 

The surface morphology of screen-printed carbon electrodes modified with reduced graphene oxide dispersed in PEDOT/PSS was characterized under SEM and compared to the morphologies of those modified with PEDOT/PSS and graphene oxide. 

**Figure 2:**
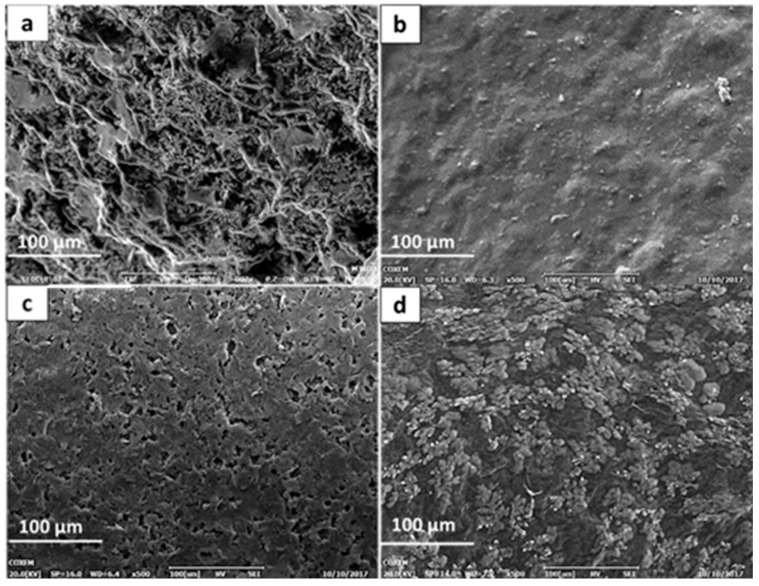
SEM images at 10x magnification of SPCE modified with (**a**) rGO, (**b**) PEDOT:PSS, (**c**) GO-PEDOT:PSS, and (**d**) rGO-PEDOT:PSS. The rGO layers appeared flaky with a discernible spacing between layers, whereas the PEDOT-PSS appeared as a well-dispersed surface of colloids. The rGO-PEDOT:PSS composite layer appeared to form a dispersion of colloidal aggregates that occupied the interlayer spacing. This dispersion appeared to be different compared to the GO-PEDOT:PSS composite layer, which may have been attributed to the different stacking characteristic of GO and rGO.

**Figure 3:**
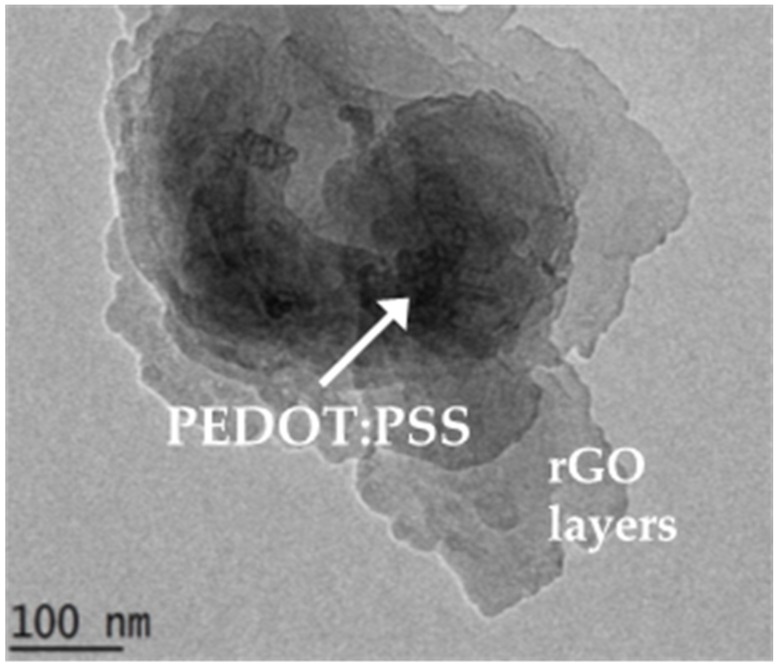
TEM image of the rGO-PEDOT:PSS composite. PEDOT:PSS appeared to be present between the rGO layers, as implied in the image by the darker impression within the rGO layers.

Reduced graphene oxide appeared to be flaky, resembling the pattern of a crumpled sheet with discernable spacing between the rGO layers (Figure 2a). This could be attributed to the re-formation of the π-π stacking between the layers as a result of electrochemical reduction [30]. With the inclusion of PEDOT:PSS into rGO (Figure 2d), the interlayer spacing of rGO appeared to be filled with PEDOT:PSS, suggesting that the π-π interaction was now taking place between the rGO and the PEDOT:PSS [31]. This was further confirmed by TEM (Figure 3). 

The TEM image shows several layers of rGO with inter-dispersion of PEDOT:PSS, which appeared as a darker layer. This shows that PEDOT:PSS had diffused into the layers of the stacking and formed the π-π interaction between PEDOT:PSS and the surface of the rGO layers [21,32]. The rGO layer image is in congruence with the images from Abdolhosseinzadeh et al. [32], and the rGO-PEDOT:PSS agrees with Chen et al. [31].

Comparing Figure 2c,d, the difference in morphology may suggest that the interaction between rGO and PEDOT:PSS was continuous and well dispersed compared to GO-PEDOT:PSS. Bundles of aggregated graphene and polymer could be seen in Figure 2d, which could be related to the PEDOT aggregation as the PSS formed the tightly coated layer with rGO.

**Figure 4:**
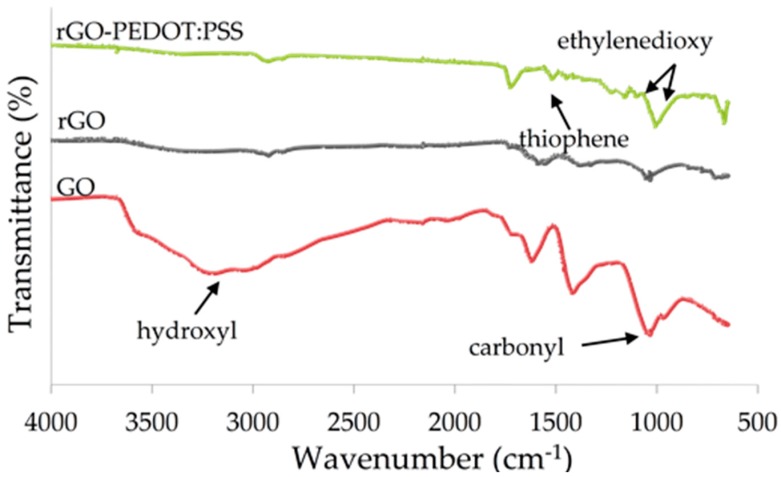
FTIR analysis of the transducer materials. The FTIR plots compare the functional-group composition between GO (red), rGO (grey), and rGO-PEDOT:PSS (green).

The FTIR results in Figure 4 show the removal of oxygenated functional groups from rGO compared to GO. The removal of the hydroxyl group was most distinct, as shown by the absence of transmittance at 3400 cm^−1^. Removal of the carbonyl groups from rGO was also suggested by the reduction in vibration at 1084 cm^−1^. This suggests that reduction of rGO could be done electrochemically on the SPCE surface. 

Comparison of the FTIR spectra of rGO and rGO-PEDOT:PSS suggests the formation of new bond types in rGO-PEDOT:PSS. The reappearance of vibration at 1090 cm^−1^ was suggested to correspond to the sulfone groups of the PSS molecules. Peaks at 1513 cm^−1^ corresponded to the C=C stretching in thiophene; stretching of the ethylenedioxy group could be seen by the vibrations at 1145 cm^−1^ and 1056 cm^−1^. 

The spectrum confirmed the reduction of graphene oxide as well as the dispersion of PEDOT:PSS in rGO. Interestingly, the band at 1726 cm^−1^, which was attributed to the graphitic C=O group, could be seen in the rGO-PEDOT:PSS spectrum [33]. This suggests that the rGO was partially reduced but sufficient enough to form the π-π interaction with PEDOT:PSS [34].

**Figure 5:**
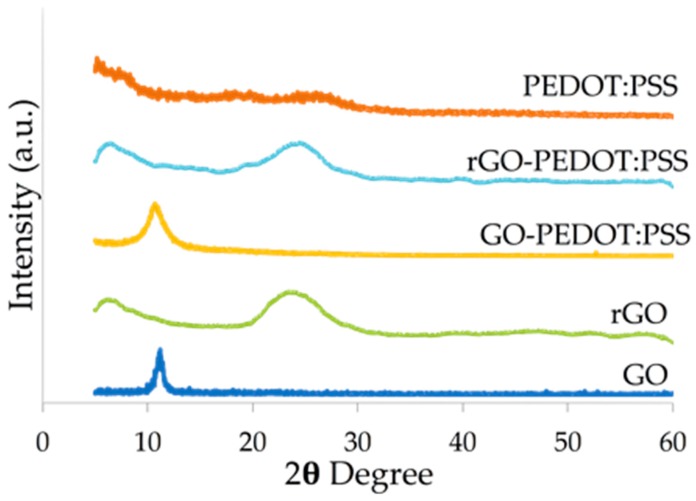
X-ray diffraction intensities of the different transducer composites. The XRD spectrum compares the diffraction between GO, rGO, GO-PEDOT:PSS, rGO-PEDOT:PSS, and PEDOT:PSS. Shift in the 2θ angle could be seen between GO and rGO in the spectra both with and without PEDOT:PSS. The peak intensities for GO and GO-PEDOT:PSS were observed at 10.7° and 10.3°, respectively, whereas for rGO and rGO-PEDOT:PSS, they were observed at 24.5° and 23.5°, respectively.

The XRD plot in Figure 5 shows a shift in the 2θ angle between the GO and the rGO. This shift confirmed the reaggregation of the rGO layers forming the π–π interaction between stacks. The reduction to rGO reestablished an sp2 carbon network that resembled that of graphene, but also caused defects to form in rGO [35]. This was shown by the broad band of the diffraction in rGO and rGO-PEDOT:PSS compared to the sharp peak in GO and GO-PEDOT:PSS. However, the XRD data confirmed only the electrochemical reduction of rGO and rGO-PEDOT:PSS and not the dispersion of PEDOT:PSS in GO and rGO. From the diffraction data, the distance between layers could be calculated by Bragg’s Law. GO and GO-PEDOT:PSS had a layer distance of 7.95 Å and 8.30 Å, respectively, whereas rGO and rGO-PEDOT:PSS had a smaller layer distance of 3.85 Å and 3.55 Å, respectively. This again reconfirmed that while GO layers were separated, rGO layers were close enough to form an interlayer interaction. It is still possible that with the inclusion of PEDOT:PSS in the matrix of rGO, the π-π interaction was formed between the surface of rGO and PEDOT:PSS [21].

**Figure 6:**
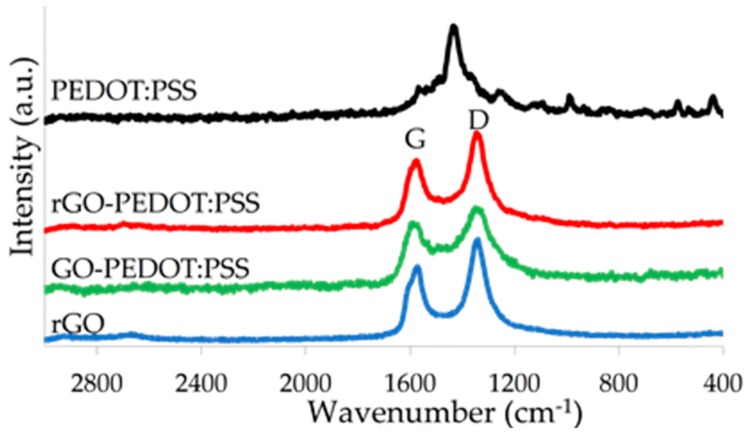
Raman spectra of rGO (blue), PEDOT:PSS (black), GO-PEDOT:PSS (green), and rGO-PEDOT:PSS (red). The D and G bands of rGO, GO-PEDOT:PSS, and rGO-PEDOT:PSS were clearly visible in the spectra, whereas the PEDOT:PSS spectrum showed a clear thiophene peak at 1434 cm^−1^. A raised intensity at 1434 cm^−1^ in both the GO-PEDOT:PSS and rGO-PEDOT:PSS spectra, compared to rGO, suggests the presence of PEDOT:PSS in the composite.

Figure 6 shows the Raman spectra of rGO, PEDOT:PSS, GO-PEDOT:PSS, and rGO-PEDOT:PSS. Two main peaks were clearly visible from the Raman spectra of all the graphene based-modified electrodes—the G (1580 cm^−1^) and D (1350 cm^−1^) bands. The higher D bands in both rGO and rGO-PEDOT:PSS compared to their G bands concurred with the expected graphitic defects of a reduced graphene oxide. The reduction process, which removed oxygen functional groups from an otherwise insulative graphene oxide, caused an increase in the out-of-plane sp2 vibrations of the carbon lattice [36]. This resulted in a higher D-band intensity in the Raman scattering. PEDOT:PSS could be clearly discerned by its characteristic vibration at 1434 cm^−1^, which was attributed to the stretching of the thiophene ring [37]. The presence of PEDOT:PSS in the rGO-PEDOT:PSS could barely be detected by a slight increase in the vibration between the D and G peaks compared to those of rGO. The weak detection here may have been due to the weak intensity obtained from the Raman data. Nonetheless, the presence of PEDOT:PSS was confirmed by other methods of detection, and the Raman data reconfirmed it, as well as showing the reduction of graphene oxide. It has been suggested that the thiophene-related band of PEDOT:PSS diminishes with respect to the graphitic D and G bands in rGO-PEDOT:PSS because PEDOT:PSS undergoes a structural change to facilitate the formation of a new π-π interaction between PEDOT and the surface of the graphitic layers [31]. This new formation forms a tight matrix within the rGO-PEDOT:PSS composite. PEDOT is known to be the molecular backbone of the conducting polymer, which acts as the hole carrier that transports charges between the valence and conduction bands [38]. In addition to the conductive behavior of rGO, inclusion of PEDOT:PSS could potentially improve conductivity of the composite. It is therefore interesting to examine the electrochemical performance of electrodes modified with rGO-PEDOT:PSS and compare it with that of rGO alone. 

### 3.2. Cyclic Voltammetric Comparison between rGO and rGO-PEDOT:PSS

**Figure 7:**
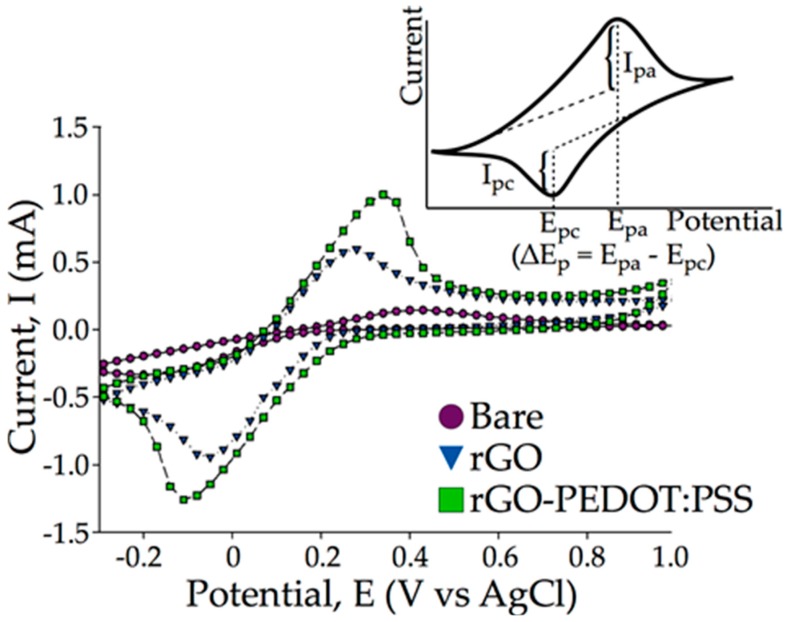
Cyclic voltammetry (CV) measurements of rGO-PEDOT:PSS/SPCE compared to those for rGO/SPCE and a bare SPCE. The peak currents for both oxidation and reduction cycles were measured as the vertical distance between the highest current peak with respect to the linear projection of the baseline of the respective curves. The anodic peak currents (I_pa_) for SPCE, rGO/SPCE, and rGO-PEDOT:PSS/SPCE were measured as 0.024 mA, 0.613 mA, and 1.005 mA, respectively. Shift in the peak potential could also be observed from the voltammogram. The shift in peak potential for each CV measurement was measured as the difference between the anodic peak potential and the cathodic peak potential, (ΔEp = E_pa_ − E_pc_). Inset figure illustrates how peak currents and peak potential shift were calculated.

Cyclic voltammetry comparison between rGO and rGO-PEDOT:PSS corroborated the characteristic contribution of PEDOT:PSS as a conducting polymer. It can be seen in Figure 7 that the peak current increased with the addition of PEDOT:PSS when comparing voltammograms of electrodes modified with rGO and with rGO-PEDOT:PSS. The result shows that the redox reaction at all electrodes in ferricyanide solution was reversible with oxidation and reduction. The rGO-PEDOT:PSS/SPCE (I_pa_ = 1.005 mA) exhibited larger current responses and better defined redox peaks towards analytes than did the rGO/SPCE (I_pa_ = 0.613 mA) and unmodified SPCE (I_pa_ = 0.024 mA). 

Modifying the electrode with rGO alone caused an increase in the peak current. This improvement in current transduction was brought about by the conductivity of the rGO, which propagated charge through its π-cloud [39]. Further incorporation of PEDOT:PSS then further increased the charge transport through the rGO-PEDOT:PSS matrix. This suggested that as the PEDOT:PSS layers were now forming the π-π interaction with rGO, electron (hole) transport along the conducting polymer contributed towards increasing the charge transfer between the electrode and the electrolyte [40]. It is likely that charge transfer was improved owing to the increased availability of π-bonding aromatic rings, which led to higher occurrence in the π-π overlap [41]. A larger number of π-π overlaps contributed to more electron delocalization around the aromatic ring, thus creating a favorable condition for electrical conductivity [42]. 

Shifts in the peak potential, E_p_, at the scan rate of 100 mV/s for SPCE, rGO/SPCE, and rGO-PEDOT:PSS/SPCE were also observed. The peak potentials were E_pa_ = 0.43 V, and E_pc_ = −0.17 V for unmodified SPCE, E_pa_ = 0.28 V and E_pc_ = −0.05 V for rGO/SPCE, and E_pa_ = 0.34 V and E_pc_ = −0.11 V for rGO:PEDOT:PSS/SPCE, respectively. The difference between the two peak potentials (ΔEp = E_pa_ − E_pc_) was calculated to be 0.6 V, 0.33 V, and 0.45 V for SPCE, rGO/SPCE, and rGO-PEDOT:PSS/SPCE, respectively. The lower potential difference for rGO/SPCE and rGO-PEDOT:PSS/SPCE indicated improved electrocatalytic ability of a modified electrode compared to an unmodified one. Overall, the addition of rGO-PEDOT:PSS to SPCEs contributed to faster electron transfer between electrode surface and electrolyte, making this composite a suitable transducer for biosensing purposes.

**Figure 8:**
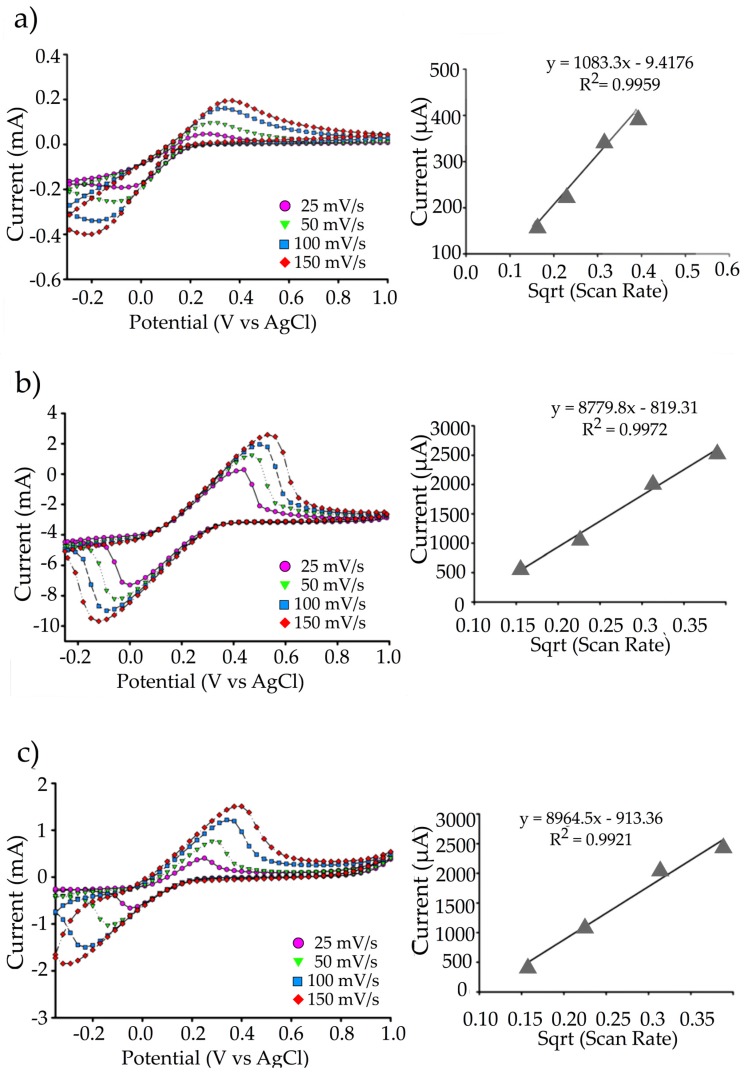
Scan-rate measurements of (**a**) SPCE, (**b**) rGO/SPCE, and (**c**) rGO-PEDOT:PSS/SPCE. Cyclic voltammetric measurements were taken at scan rates of 25, 50, 100, and 150 mV/s. Linear regression plots were derived from the scan-rate voltammogram by plotting the corresponding peak currents and potentials at each scan rate.

Figure 8 shows the individual voltammograms of the SPCE, rGO/SPCE, and rGO-PEDOT:PSS/SPCE in potassium ferricyanide (K_3_Fe(CN)_6_) solution at different scan rates (25, 50, 100, and 150 mV/s) and their corresponding linear regression plots. The results show that the current and peak potential increased linearly with the scan rate for all three electrode types. This suggests that the redox current of all electrode types in ferricyanide solution was dependent on the scan rate. In addition, the effective surface area was also calculated based on the Randles-Sevcik equation. The calculated effective surface areas for SPCE, rGO/SPCE, and rGO-PEDOT:PSS/SPCE were 1.6 mm^2^, 12.6 mm^2^ and 12.9 mm^2^, respectively. The large increase in effective surface area of the rGO/SPCE and rGO-PEDOT:PSS/SPCE in comparison to the SPCE could be attributed to the increase in the charge transferability and current density. The slight increment between rGO/SPCE and rGO-PEDOT:PSS/SPCE effective surface area, on the other hand, could be related to the tight layer of the PEDOT:PSS aggregates occupying the interlayer spacing between the rGO surfaces [21,31,38]. 

### 3.3. rGO-PEDOT:PSS as a Transducer in Enzymatic Sensing

Suitability of the rGO-PEDOT:PSS composite for use in enzymatic-based sensors was then tested. Glucose oxidase (GOx) was used as our model enzyme because its use is well established in glucose biosensors, and its mechanism of reaction is known [43]. Glucose oxidase was incorporated into the transducer composite to form a homogeneous biorecognition-transducer complex (rGO-PEDOT:PSS-GOx). 

**Figure 9:**
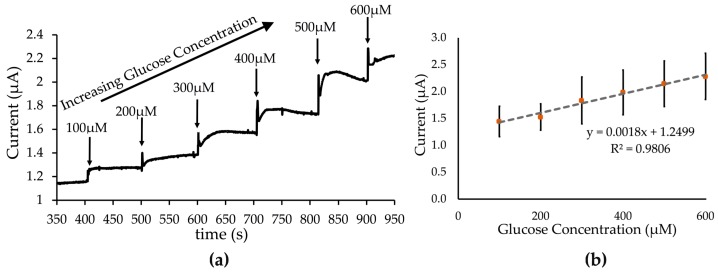
Amperometric titration of glucose concentration measured at a single step potential of 0.5 V. (**a**) Amperometric current-time measurement of glucose detection using electrodes modified with rGO-PEDOT:PSS-GOx. Glucose concentrations were increased incrementally in 100-s time intervals. (**b**) Calibration curve derived from the amperometric measurements taken from SPCE electrodes modified with rGO-PEDOT:PSS (n = 3). The plots were fitted linearly throughout the measured glucose range (100 µM–600 µM) with an R-squared value of 0.9806.

Figure 9a shows a sample of the amperometric current versus time curve of glucose introduced onto the rGO-PEDOT:PSS-GOx electrode. Incremental addition of glucose onto the electrode resulted in a stepwise increase in current. This suggests that the electrode could detect changes in glucose concentration within the range of 100 µM to 600 µM. This is also reflected by the linear plot throughout the calibration curve in Figure 9b. From here, the sensitivity of the glucose detection was obtained as 57.3 µA/(mM·cm^2^) and its limit of detection at 86.8 µM glucose, per standard calculation [44,45]. The sensitivity and limit of detection of this initial setup appeared to be within an acceptable range compared with those of other modified electrodes [46]. 

Table 1 compares the limit of detection of several glucose oxidase-based sensors. Notably, modifying the SPCE with ionic liquid was shown to improve its limit of detection significantly [47]. However, limited access to this method could possibly limit rapid prototyping and mass fabrication of biosensors. Previous work has also reported lower limits of detection of other graphene-based glucose sensors [48]; however, these sensors are mostly based on glassy carbon electrodes. Screen-printed carbon electrodes appeal to the development of biosensors owing to their cost-effectiveness and ease of miniaturization. 

Nevertheless, refinements can be pursued in improving our glucose detection using rGO-PEDOT:PSS as the transducer. This is particularly true for improvising ways to include glucose oxidase or other enzymes that can improve methods in detecting glucose [11], as well as translating its signal through the rGO-PEDOT:PSS transducer. 

## 4. Conclusions

Modification of commercially cheap screen-printed carbon electrodes brings an attractive alternative that allows us to improve the transfer kinetics of SPCEs. We have shown here that PEDOT:PSS forms a tight matrix that is interspersed between the rGO layers. This allows PEDOT to form a π-π interaction with the surface of rGO, which could improve conductivity. This work also shows that modifying the electrode surface with a composite of reduced graphene oxide and PEDOT:PSS (rGO-PEDOT:PSS) can improve signal transduction at the electrolyte-electrode interface. It is shown that graphene oxide can be electrochemically reduced on an SPCE. The use of rGO-PEDOT:PSS is electrochemically better than the use of rGO alone. Preliminary work has also demonstrated that glucose oxidase can be incorporated into the composite matrix and detect changes in glucose concentration. It is possible to explore this potential further by either employing other preparation strategies or using other enzymes, which could improve detection.

## Figures and Tables

**Table 1:** Sensitivity and limit of detection comparison of a few selected glucose enzymatic sensors (glucose oxidase). PVDF: polyvinylidene, µPAD: paper-based analytical device, 4-APBA: 4-aminophenylboronic acid, NRs: nanorods, ITO: indium tin oxide, Pt: platinum, PANI: polyaniline, AuNPs: gold nanoparticles, Gr: graphene, IL: ionic liquid, N/D: not described.

	Electrode	Method	Sensitivity (µA/(mM·cm^2^))	LoD (mM)	References
1	GOx/rGO/IL- SPE	CV	22.78	0.001	[47]
2	PVDF/Ag/GOx	Capacitive	N/D	13	[49]
3	(µPAD)GOx/4-APBA/cellulose-modified SPCE	CV	N/D	0.86	[50]
4	Nafion/GOx/ZnO NRs/ITO	CV	48.75	0.06	[51]
5	GOx/Pt-graphite SPE	CV	105	0.01	[52]
6	Gr/PANI/AuNPs/GOx/SPCE	DPV	20.32	0.1	[53]
7	rGO-PEDOT:PSS-GOx/SPCE	CV	57.3	0.0868	This work
